# Binding of Human Milk to Pathogen Receptor DC-SIGN Varies with Bile Salt-Stimulated Lipase (BSSL) Gene Polymorphism

**DOI:** 10.1371/journal.pone.0017316

**Published:** 2011-02-28

**Authors:** Martijn J. Stax, Marloes A. Naarding, Michael W. T. Tanck, Susanne Lindquist, Olle Hernell, Robert Lyle, Per Brandtzaeg, Merete Eggesbø, Georgios Pollakis, William A. Paxton

**Affiliations:** 1 Laboratory of Experimental Virology, Department of Medical Microbiology, Center for Infection and Immunity Amsterdam (CINIMA), Academic Medical Center, University of Amsterdam, Amsterdam, The Netherlands; 2 Department of Clinical Epidemiology, Biostatistics and Bioinformatics, Academic Medical Center, University of Amsterdam, Amsterdam, The Netherlands; 3 Pediatrics Unit, Department of Clinical Sciences, Umeå University, Umeå, Sweden; 4 Department of Medical Genetics, Oslo University Hospital, Oslo, Norway; 5 LIIPAT, Centre for Immune Regulation, University of Oslo, and Department of Pathology, Oslo University Hospital, Rikshospitalet, Oslo, Norway; 6 Division of Epidemiology, Department of Genes and Environment, Norwegian Institute of Public Health, Oslo, Norway; University of California San Francisco, United States of America

## Abstract

**Objective:**

Dendritic cells bind an array of antigens and DC-SIGN has been postulated to act as a receptor for mucosal pathogen transmission. Bile salt-stimulated lipase (BSSL) from human milk potently binds DC-SIGN and blocks DC-SIGN mediated *trans*-infection of CD4^+^ T-lymphocytes with HIV-1. Objective was to study variation in DC-SIGN binding properties and the relation between DC-SIGN binding capacity of milk and BSSL gene polymorphisms.

**Study Design:**

ELISA and PCR were used to study DC-SIGN binding properties and BSSL exon 11 size variation for human milk derived from 269 different mothers distributed over 4 geographical regions.

**Results:**

DC-SIGN binding properties were highly variable for milks derived from different mothers and between samplings from different geographical regions. Differences in DC-SIGN binding were correlated with a genetic polymorphism in BSSL which is related to the number of 11 amino acid repeats at the C-terminus of the protein.

**Conclusion:**

The observed variation in DC-SIGN binding properties among milk samples may have implications for the risk of mucosal transmission of pathogens during breastfeeding.

## Introduction

Human milk contains a large array of foreign antigens and host factors that interact with esophageal and gut mucosa of the breastfed infant [1], [2]. There is accumulating evidence that breast milk protects the newborn against infections as indicated by the decreased morbidity and mortality due to diarrhea among breastfed as compared to formula-fed infants [1], [3]. Furthermore, only 4–12% of human immunodeficiency virus type-1 (HIV-1) positive mothers transmit HIV-1 to their infant during breastfeeding in spite of frequent exposure of the infant to HIV-1 positive breast milk for up to several years [4]. If we would have a better understanding of how human milk influences mucosal infections, we would be able to develop therapeutics aimed at prevention.

The anti-microbial activity of human milk is, at least in part, the result of secretory antibodies and prebiotic factors. Glycans in breast milk have been associated with protection against transmission of specific mucosal pathogens [1], [5]. Furthermore, blood group antigen genes involved in post translational protein modification and resulting in differentiated glycan fingerprints have been linked to pathogen driven selection in humans [6]. This stresses the relevance of glycans in the continuous competition with rapidly evolving pathogens where one glycan type may protect against certain pathogens whilst enhance infection with other pathogens. Although innate immune molecules present in breast milk can contribute to protection against infection, additional immune responses need to be activated in the infant for further effector and memory purposes. Antigen presenting cells such as dendritic cells (DCs) that capture invading pathogens through pathogen coat sugars regulate such processes.

Pathogen receptor DC-Specific Intercellular adhesion molecule-3 Grabbing Non-integrin (DC-SIGN), also known as CD209, is highly expressed in mucosal tissues and binds a wide range of pathogens such as HIV-1, *Helicobacter pylori* and *Candida albicans* [7]–[10]. Such interaction between pathogen and DC-SIGN expressing cells could occur at damaged epithelia. Normally, invading pathogens captured by DC-SIGN expressing DCs are degraded and the processed antigens are subsequently presented to the appropriate T-cells. Although the capture and presentation of invading pathogens is essential for inducing adaptive immune responses, some pathogens escape from full degradation and in fact hijack this system to enhance infection [7], [11]. The biological relevance of DC-SIGN in transmission of HIV-1 and *Mycobacterium tuberculosis* is supported by linkages between DC-SIGN polymorphisms and risk of infection [12], [13]. We previously reported that bile salt-stimulated lipase (BSSL) from human milk strongly binds to DC-SIGN and interferes with DC-SIGN mediated HIV-1 transmission *in vitro* [14], [15]. Interestingly, BSSL has also been associated with protection against Norwalk virus infection, which is a major cause of gastroenteritis [5], [16]. These studies indicated two possible anti-microbial mechanisms for BSSL, either through binding to DC-SIGN (HIV-1) [15] or binding to the virus particle (Norwalk virus)[5].

BSSL is a lipase that is present in human blood and breast milk which aids breastfed infants with the digestion of milk triglycerides [17], [18]. Tissue specific BSSL expression in mice is influenced by nuclear factor 1-C2 (NF1-C2) and up-regulation of the BSSL gene is correlated with an increase in the number of differentiated epithelial cells [19], [20]. The human BSSL glycoprotein contains a mucin-like repeated 11 amino acid motif at the C-terminal tail that is abundantly modified by O-linked glycosylation. The repeated motif, encoded by exon 11 of the gene does not play a role in the enzymatic activity of the lipase [21], [22]. Furthermore, the O-glycosylated repeated motif expresses Lewis a and Lewis x sugars known to interact with DC-SIGN [23], [24]. We previously described that BSSL binds to DC-SIGN via Lewis x sugars. Furthermore we suggested that variation in BSSL protein size among mothers may relate to variation in DC-SIGN blocking capacities [15].

The aim of this study was to characterize the DC-SIGN binding properties of breast milk derived from healthy breastfeeding mothers. Furthermore, we compared BSSL size variations with DC-SIGN binding capacity of breast milks. Our results reveal that the DC-SIGN binding capacity of breast milk is highly variable among mothers as well as among groups of mothers from differing geographical regions. We were able to correlate allelic polymorphisms in the BSSL gene with the DC-SIGN binding phenotype of breast milk. The observed variation in DC-SIGN binding properties of different milks may have implications for the risk of mucosal pathogen transmission during breastfeeding.

## Results

### BSSL protein size is linked to DC-SIGN binding capacity of the corresponding human milk

**Figure 1:**
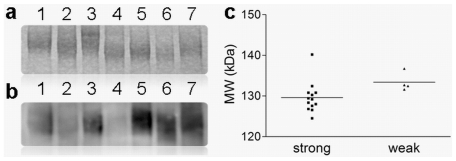
DC-SIGN binding is highly variable and correlates with BSSL protein size. (a) Example of a coomassie stained SDS-PAGE separation of breast milk from 7 mothers showing the bile salt-stimulated lipase (BSSL) of variable sizes. (b) Western blot stained with DC-SIGN-Fc of the same 7 milks as depicted in figure a. (c) Breast milks with smaller BSSL protein have stronger DC-SIGN binding capacity than breast milks with larger BSSL protein. Molecular weights (MW) of BSSL protein was compared in milks with strong DC-SIGN binding capacity versus milks with weak DC-SIGN binding capacity. Median protein sizes in the weak and in the strong DC-SIGN binding groups are indicated by a horizontal line.

We have previously identified BSSL from human milk as a strong DC-SIGN binding glycoprotein with variably sized BSSL isoforms differing in their capacity to bind DC-SIGN [15]. We hypothesized that the DC-SIGN binding capacity of BSSL and human milk is correlated with the BSSL protein size. To test this hypothesis, we determined the BSSL protein size and DC-SIGN binding capacity of human milk for 17 mothers from the Netherlands. BSSL protein size was estimated for breast milk separated by SDS-PAGE (figure 1a). The DC-SIGN binding capacity of milk was detected by Western blot (figure 1b) and quantified for all milk samples by ELISA (figure 1c). DC-SIGN binding capacity was highly variable (figure 1b+c) whereas BSSL expression levels were similar in the milks for which BSSL protein size was determined. Although, some milks (not included in this analysis) had decreased BSSL expression levels these cases did not correlate to decreased DC-SIGN binding properties of the corresponding milks. Figures 1a and b represent an example of milks from different mothers with similar BSSL expression levels but variable DC-SIGN binding capacities.

We calculated the relative DC-SIGN binding capacity with the strongest DC-SIGN binding milk arbitrarily set to 100%. We observed a cluster of 4 samples with weak DC-SIGN binding properties deviating from the other stronger DC-SIGN binding milk samples. We then compared BSSL protein sizes of these weak DC-SIGN binding milks (≤20% binding) to strong DC-SIGN binding milks (≥34% binding). Figure 1c shows that the BSSL protein size was significantly smaller (p = 0.020) in the strong DC-SIGN binding group than in the weak DC-SIGN binding group. However, linear regression analysis of protein size versus DC-SIGN binding capacity did not show a significant deviation.

### DC-SIGN binding capacity of human milk is highly variant within and between different cohorts

**Figure 2:**
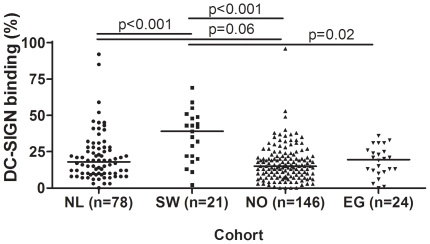
DC-SIGN affinity of breast milk is highly variable between mothers within cohorts and between different cohorts. The DC-SIGN binding of milks from the Netherlands (NL), Sweden (SW), Norway (NO) and Egypt (EG) is depicted as a percentage of the highest overall binding sample (100%). Median DC-SIGN affinity is indicated by a horizontal line for each cohort.

After identifying variation in DC-SIGN binding capacity of breast milk derived from a group of mothers from the Netherlands, we investigated the level of variation within and between different cohorts. We therefore tested DC-SIGN binding of human milk derived from the Netherlands (n = 78), Sweden (n = 21), Norway (n = 146) and Egypt (n = 24) using ELISA. Figure 2 shows the DC-SIGN binding of all breast milk samples with the milk having the strongest DC-SIGN binding set to 100%. These results show that DC-SIGN affinity of human milk is highly variable among mothers in all four cohorts. In addition, significant differences were apparent between the cohorts. The breast milk samples from Sweden bound significantly stronger to DC-SIGN than the milks from the other cohorts (figure 2). Breast milk samples from the Netherlands had a marginally stronger (p = 0.061) DC-SIGN binding than the tested samples in the Norwegian cohort.

### The size of BSSL exon 11 is highly polymorphic

**Figure 3:**
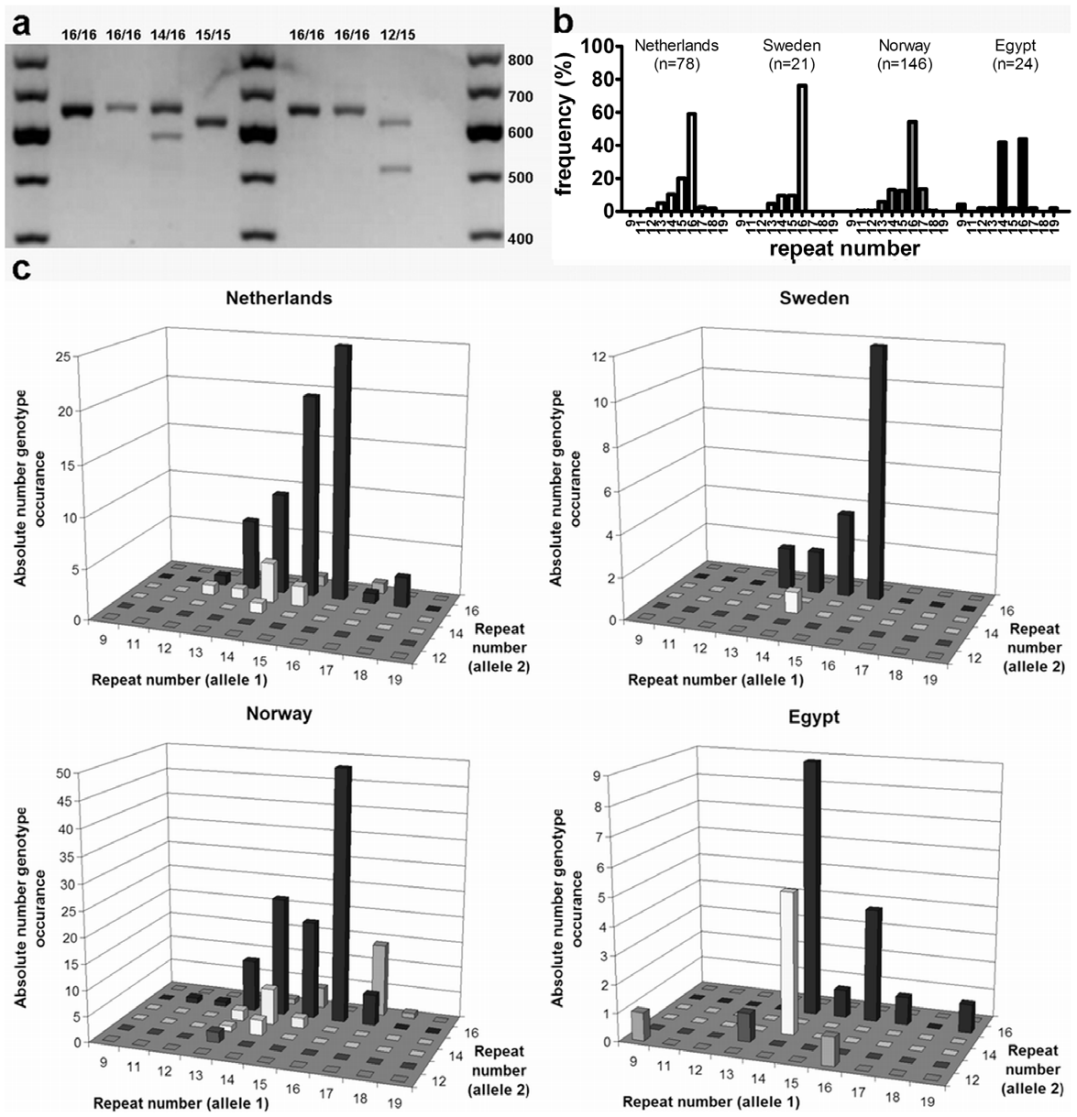
BSSL exon 11 is highly polymorphic in number of repeats. (a) Typical agarose gel analysis of BSSL exon 11 PCR genotyping including 3 marker lanes and 7 genotyped DNA samples. (b) Allelic repeat number variation as determined by PCR analysis within each cohort. Repeat number frequency is indicated as percentage of the total number of alleles within a cohort. (c) BSSL genotype distribution is highly variable in all cohorts with most mothers having at least 1 allele with 16 repeats. Overview is plotted of the BSSL repeat number genotype combinations per milk sample within each cohort.

BSSL protein size variation may be related to variation in the variable number of tandem repeats (VNTR) domain encoded by BSSL exon 11. BSSL binds to DC-SIGN through Lewis type sugars present in this VNTR domain that is located at the C-terminus of the protein [15], [23]. We established a PCR to determine the size of the VNTR domain (figure 3a) and subsequently estimated the possible number of repeats in each allele. The VNTR domain is highly variable in the number of repeats in the populations we tested, with repeat numbers ranging from 9 to 19 and alleles with 16 repeats being most common (figure 3b). The allele frequency distribution in the Egyptian cohort differs significantly from the European cohorts (p<0.001). Additionally, the allele frequency distribution in the Norwegian mothers differs significantly from that in Dutch mothers (p<0.001) and marginally (p = 0.074) from the Swedish mothers. When looking at the genotype distribution in figure 3c we observed that 79% of all mothers (82% in European cohorts) had at least 1 allele with 16 repeats.

### DC-SIGN binding capacity correlates to BSSL genotype

**Figure 4:**
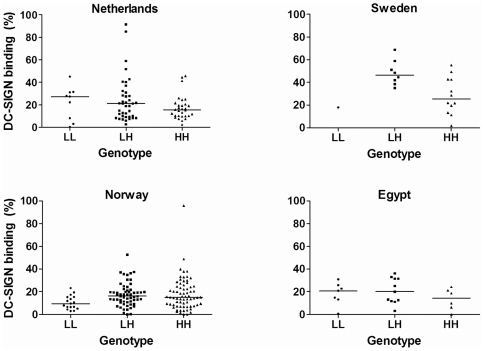
DC-SIGN binding capacity correlates to BSSL genotype. Alleles are defined as L (9 to 15 repeats) or H (16–19 repeats). The BSSL genotype is plotted against DC-SIGN affinity for mothers from the Netherlands, Sweden, Norway and Egypt. LL, LH and HH genotypes are plotted against DC-SIGN binding. Median DC-SIGN binding within cohorts is indicated by a horizontal line.

Differences in BSSL protein size were linked to the DC-SIGN binding properties of milk (figure 1c). In addition, we hypothesized that variation in DC-SIGN binding may be caused by size differences in the VNTR domain of the BSSL gene. To test this possibility we compared VNTR domain sizes with DC-SIGN binding potency of the corresponding milks. The majority of tested mothers have at least one allele with 16 repeats whereas the non-16 repeat BSSL variants mostly have less than 16 repeats (see figure 3b). We arbitrarily defined less than 16 repeats as low (L) and 16 or more repeats as high (H) repeat number. Mothers have either two low (LL), one allele with low and one with high (LH) or two alleles with high repeat numbers (HH). The BSSL repeat number has a significant (p = 0.018) effect on the DC-SIGN binding capacity for all 4 tested cohorts (figure 4a). In the 3 European cohorts we found that mothers with the LH genotype had significantly stronger DC-SIGN binding milk (p = 0.016) than mothers with the HH genotype. These LH mothers also had marginally significant stronger DC-SIGN binding milk (p = 0.064) than mothers with the LL genotype. The LL genotype reaches low frequencies, as the majority of individuals carry at least 1 allele with high repeat numbers (figure 3c). The H-repeat group (n = 353) mainly exists of 16 repeat BSSL alleles (n = 303) and only 50 alleles having more than 16 repeats. Alleles with more than 16 repeats are mainly found in the Norwegian population (n = 41).

## Discussion

In this study we demonstrate that the DC-SIGN binding capacity of human milk from different mothers is highly variable. We confirmed this finding in 4 independent cohorts and report that variation exists between the samplings from different geographical regions in the DC-SIGN binding capacity. BSSL genes have either a high number of repeats (H = 16 to 19) or a low number of repeats (L = 9 to 15) in the VNTR domain. We observed that the combination of an L allele with an H allele (LH) correlates with strong DC-SIGN binding of breast milk. In addition, for mothers with at least one BSSL allele of 16 repeats we report that strong DC-SIGN binding is correlated with a small repeat number (16+L) in the second allele.

The observed variation in DC-SIGN binding capacity may in theory be related to diverse factors such as the BSSL expression level or polymorphisms in the BSSL gene. Analysis of DC-SIGN binding variation in breast milk samples with similar BSSL protein levels demonstrates that factors other than BSSL protein concentration determine the DC-SIGN binding efficiency. Although we showed that BSSL polymorphisms are correlated with DC-SIGN binding we do not exclude that in case of breast milk samples with significantly deviating BSSL protein levels DC-SIGN binding can be influenced. We previously reported that BSSL is the major DC-SIGN binding component in human milk, but mucin 1 - mainly present in milk lipid vesicles - also binds to DC-SIGN [25]. However, we measured DC-SIGN binding of the aqueous phase of breast milk, which contains the BSSL glycoprotein and excludes the lipids [15], [25]. We therefore believe that BSSL and not mucin 1 is the main source of the DC-SIGN binding we measured.

DC-SIGN forms tetramers with four binding pockets that have higher affinity for glycoproteins such as HIV gp120 than monomeric DC-SIGN [26]. It therefore seems likely that glycoproteins carrying multiple DC-SIGN interacting sugars will have a higher affinity for DC-SIGN than glycoproteins with less of such glycans. Since BSSL is polymorphic in the number of potential O-glycosylation sites due to variation in the VNTR domain this might translate into increased affinity of long BSSL forms for DC-SIGN. In contrast, we initially found that breast milks with small BSSL proteins have a stronger DC-SIGN binding capacity than breast milk with large BSSL proteins. Our genotyping studies showed that the LH genotype is associated with stronger DC-SIGN binding than the LL and the HH genotype. But the frequency of the LL genotype is low and gene size variation may not always correlate with BSSL glycoprotein size. The size of the processed glycoprotein may vary due to differences in glycosylation among different individuals and also truncated forms have been reported [27]. However, our genotyping data may suggest that BSSL forms dimers and that the combination of a low with a high repeat molecule provides the combination with optimal DC-SIGN binding properties. This is supported by the observation of McKillop and colleagues who observed that purified BSSL protein forms dimers under native conditions between either two equally or differently sized BSSL proteins [28].

We speculate that not only the number of glycans could play a role, but additionally other factors likely influence the DC-SIGN binding properties of BSSL such as the three dimensional structure of the protein. We propose that the quaternary structure of the BSSL dimer complex plays an additional role in determining the DC-SIGN binding properties by influencing the way BSSL sugars are presented to DC-SIGN. The combination of a high and low repeat BSSL protein is possibly presenting the best combination of a high number of DC-SIGN interacting glycans with an optimal three dimensional structure for binding to DC-SIGN.

Not all O-linked BSSL glycans carry DC-SIGN binding Lewis sugars [23] and the number of Lewis sugars per BSSL molecule may be influenced by variable activity of glycosidases [16], [29]. The activity of such enzymes may add an additional level of complexity to the observed link between BSSL genotypes and DC-SIGN binding capacity of breast milk and enzyme expression levels may vary over time during lactation as can the post-translational glycosylation profiling which may also influence strength of binding [30]. Specifically secretors, mothers that carry functional fucosyltransferase 2 (FUT2) genes, may express different Lewis type sugars than mothers deficient for FUT2 (non-secretors) [5]. Such differences as well as polymorphisms in other glycosylating genes may well influence the number and type of DC-SIGN binding glycans present on the BSSL glycoprotein. This extra layer of complexity in glycoprotein expression may help explain the different behavior of LL genotyped breast milks with regard to DC-SIGN affinity in the Norwegian population versus Dutch population. However, care should be taken because of the relatively low number of LL genotyped mothers (n = 9) in the Dutch cohort and the high level of variation in DC-SIGN binding observed in this group. Cloning and expression of recombinant BSSL forms in cell lines should aid in further clarifying observed differences in DC-SIGN binding between BSSL proteins with variably sized repeat motifs.

Pathogen binding to DC-SIGN results in uptake by DCs and subsequent antigen presentation to T-cells but this mechanism appears to have been hijacked by some pathogens to promote their transmission [7], [31]. In addition to antigen presentation, pathogen binding by DC-SIGN triggers signal transduction resulting in modulation of DC immune activation status. DC-SIGN binding of Lewis type or high mannose glycans results into two separate routes of signaling depending on the glycan bound [32]. Human milk, semen and cervicovaginal secretions contain factors that interfere with the interaction between DC-SIGN and the pathogen *in vitro* [14], [33], [34]. *In vivo* such secreted factors may reach DCs through breaches in the mucosal epithelial layer similar to how HIV-1 or other pathogens may reach DCs in the sub-epithelia We therefore suggest that the DC-SIGN blocking activity of human secretions such as milk may partially help explain why the risk of HIV-1 transmission via breastfeeding or sex is relatively low. Furthermore, the interaction between breast milk and DC-SIGN may influence the immune activation levels of DCs and other immune cells during breastfeeding. We observed a high level of variation in the DC-SIGN blocking properties of breast milk from different mothers. For the infant this variation could result in differential immune activation levels and differences in the risk of infection, depending on the breastfeeding mother.

Although blocking the interaction between pathogen and DC-SIGN may be beneficial against DC-SIGN mediated transmission, receptor availability may still be necessary for inducing effective adaptive immune responses against other pathogens. Such conflicting roles for DC-SIGN may explain why the observed variation in DC-SIGN binding properties of human milk may be beneficial at the population level. Exposure to pathogens may result in selective pressures in the direction of either strong or weak DC-SIGN blocking depending on the type of circulating pathogens dominating a specific population. We therefore speculate that the observed geographical variation in DC-SIGN binding may be a result of local selective pressures exerted over an evolutionary timescale. However, care should be taken when interpreting the observed geographical differences due to the relatively low numbers of individuals in the Swedish and Egyptian samplings. We suggest that variation in DC-SIGN blocking properties of mucosal secretions might be a general theme in natural protection against rapidly evolving mucosal pathogens. Therefore we speculate that the identified BSSL size variation may represent one of multiple strategies for a population to have variable levels of protection against mucosal pathogens, as part of innate immunity.

## Methods

### Ethics statement

Medical ethical clearance was granted by the regional ethics committee for medical research in Norway (for Norwegian samples) and the ethics committee of the medical faculty of Umea University (for Swedish samples) and with written informed consent provided. Samples collected in the Netherlands and Egypt were anonymous, single time-point collections of materials to be discarded and therefore did not require medical ethical clearance.

### Processing of human milk

Human milk was collected from 269 breastfeeding mothers from Sweden (n = 21), Norway (n = 146), The Netherlands (n = 78) and Egypt (n = 24) and with no known timing with regards to the age of the infants. The mothers were recruited from the general population and do not necessarily represent mothers of the countries from where the samples were taken. The milk was centrifuged at 530 x g for 30 min at 4°C. The fat layer on top was removed and the skim milk was collected to be used in DC-SIGN binding experiments. The remaining cell pellet was resuspended in L6 lysis buffer and the DNA was purified as previously described [35].

### SDS-PAGE and Western blot analysis

Processed human milk (skim milk) proteins were separated using 8% SDS-PAGE gels (Bio-Rad, Veenendaal, The Netherlands). Gels were stained with Coomassie stain and the BSSL protein size was estimated from gel. For immunoblotting the gels were blotted on polyvinylidene difluoride membranes (PVDF-F; Millipore, USA) and stained with DC-SIGN-Fc (R&D systems, Inc., England), and goat anti-human IgG1 antibody (Jackson Immunoresearch, USA). Visualization was performed using Odyssey infrared imaging system (LI-COR Bioschiences, USA).

### DC-SIGN binding ELISA

ELISA plates were coated with human milk 100 fold diluted in 0.2 M NaHCO_3_ buffer. Plates were blocked with 5% BSA and subsequently incubated with a recombinant human DC-SIGN-Fc chimera (R&D systems, Abingdon, England) in TSM buffer (20 mM TRIS, 150 mM NaCl, 1 mM CaCl_2_ and 2 mM MgCl_2_) containing 5% BSA as previously described [15], [33]. Peroxidase labeled anti human Ig-Fc antibodies (Jackson Immunology, Baltimore, USA) were used to quantify the bound DC-SIGN-Fc. Non specific binding of DC-SIGN-Fc was determined for each individual sample by pre-incubating the calcium dependent DC-SIGN-Fc for 20 min with 20 mM EGTA(Sigma-Aldrich, Zwijndrecht, The Netherlands).

### Genotyping PCR

Lidberg and colleagues previously identified the carboxyl ester lipase-like gene (CELL, genbank accession number M94580) with high homology to the BSSL gene [36]. CELL contains a VNTR domain resembling the exon 11 VNTR domain of BSSL although Lidberg and colleagues reported the CELL VNTR to contain 7 repeats less than BSSL. Interference of CELL during the PCR amplification of BSSL was prevented by designing primers that indisputably only amplify BSSL exon 11 and not the CELL VNTR. Primers (forward primer: ACCAACTTCCTGCGCTACTGGACCCTC, reverse primer: TGATACCAAGGCTCATGGGACGCTAAAAC) contained a FAM label for detection (Eurogentec, Maastricht, The Netherlands). PCR was performed with Reddy Mix PCR master mix (ABgene, Epsom, UK) according to the suppliers' standard protocol with betain (Sigma, St Louis, USA) added to a final concentration of 1.5 M. After initial denaturation for 4′ at 94°C the following program was run for 35 cycles: 30″ 94°C, 3′ 60°C, 1′ 72°C followed by an extended elongation for 7′ at 72°C. The PCR product was mixed with the Genescan™ –1200 LIZ® Standard (Applied Biosystems, Foster city, USA) and the ABI 3100 capillary sequencer (Applied Biosystems, Foster city, USA) in Genescan mode was used for PCR product size determination. Genemapper software (Applied Biosystems, Foster city, USA) was used to analyze the data.

### Statistical analysis

BSSL protein sizes are presented as individual observations. Average values of triplicate DC-SIGN binding capacity measurements were plotted. Median values are indicated in the figures. A Mann-Whitney test was used to compare BSSL protein size of the weak and strong DC-SIGN binding capacity groups. The Kruskal-Wallis test, with subsequent pairwise Mann-Whitney tests in case of an overall significant difference was used to analyze differences in DC-SIGN binding capacity between cohorts. Fisher-Exact test was used to compare allele frequencies between the cohorts. A non-parametric ANOVA (rank transformed values), with adjustment for cohort and the cohort*genotype interaction was used to analyze the effect of the genotype on DC-SIGN binding capacity. SPSS (release 17, SPSS Inc., USA) was used for all analyses and p-values <0.05 were considered statistically significant.
